# Cholesterol Management in Neurology: Time for Revised Strategies?

**DOI:** 10.3390/jpm12121981

**Published:** 2022-11-30

**Authors:** Felicia Liana Andronie-Cioară, Anamaria Jurcău, Maria Carolina Jurcău, Delia Carmen Nistor-Cseppentö, Aurel Simion

**Affiliations:** 1 Department of Psycho-Neuroscience and Rehabilitation, Faculty of Medicine and Pharmacy, University of Oradea, 410073 Oradea, Romania; 2 Faculty of Medicine and Pharmacy, University of Oradea, 410073 Oradea, Romania

**Keywords:** statins, cholesterol, lipid rafts, myotoxicity, myasthenia gravis, muscular dystrophies, cognitive impairment

## Abstract

Statin therapy has been extensively evaluated and shown to reduce the incidence of new or recurrent vascular events, ischemic stroke included. As a consequence, each published guideline pushes for lower low-density cholesterol levels in the population at large, recommending increased statin doses and/or adding new cholesterol-lowering molecules. Neurologists find it sometimes difficult to apply these guidelines, having to confront situations such as (1) ischemic strokes, mainly cardioembolic ones, in patients with already low LDL-cholesterol levels; (2) myasthenic patients, whose lifespan has been extended by available treatment, and whose age and cholesterol levels put them at risk for ischemic stroke; (3) patients with myotonic dystrophy, whose disease often associates diabetes mellitus and heart conduction defects, and in whom blood cholesterol management is also not settled. As such, further trials are needed to address these issues.

## 1. Introduction

Since the discovery of mevastatin (ML-236B) produced by *Penicillium citrinum* [1], the class of drugs generically known as statins have become increasingly prescribed for the prevention of vascular events due to high total cholesterol and elevated low-density lipoprotein (LDL) cholesterol levels starting with the release of the results of the Scandinavian Simvastatin Survival Study (4S) in 1994 [2]. Subsequent studies reinforced these findings [3], and guidelines recommended more and more aggressive statin treatment in an attempt to reduce cardiovascular morbidity and mortality [4,5]. As a consequence, statins, mainly atorvastatin and simvastatin, have become among the most widely prescribed drugs worldwide [6].

Adding newer agents, such as ezetimibe or proprotein convertase subtilisin/kexin type 9 (*PCSK9*) inhibitors (evolocumab, alirocumab), allows for further reduction of the serum LDL-cholesterol levels and of the risk of vascular events [7,8].

However, although efficient in reducing cardiovascular risk, statins have a series of myotoxic and cognitive side effects which can make a choice and proper dosing of statins in elderly stroke patients at risk for/with cognitive impairment or in patients with muscular dystrophies or myasthenia gravis, who have also increased cholesterol levels, a challenging task.

## 2. Cholesterol and Its Functions

Cholesterol is a basic constituent of all membranes [9] and has a pivotal role in vesicle formation and endo- as well as exocytosis, production and utilization of lipoproteins [10], and influences all important characteristics of cellular and subcellular membranes such as membrane fluidity and permeability [11], or activity of membrane receptors and ion channels [12,13]. As such, interfering with the biosynthesis of cholesterol may change the form and function of all membranes in the body [14].

**Figure 1:**
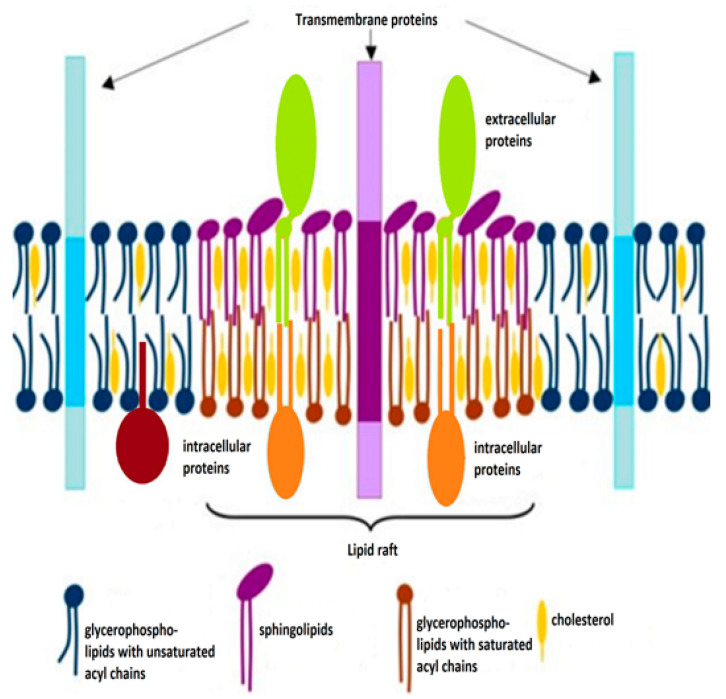
A simplified model of lipid rafts in cell membranes. Cholesterol is abundant in the lipid raft microdomain, where the acyl chains of lipids are generally longer and saturated, as opposed to the acyl chains of non-raft phospholipids, which are unsaturated and shorter.

Research has shown that cholesterol in cellular membranes forms lipid rafts (Figure 1) [15], membrane microdomains that concentrate and segregate proteins in the bilayer plane [16] and in which cholesterol acts as a "glue" holding the various domains together [17]. From a normal cholesterol content of approximately 20% of membrane lipids [14], it increases to nearly 50% in the lipid raft areas [14], which regulate membrane trafficking, a series of cell signaling cascades, as well as cell migration [18]. Figure 1. shows the distribution of cholesterol in these lipid rafts.

In the nervous system, the cholesterol content is even higher, with about 30% being included in membranes and 70% of the nervous system’s cholesterol content being used by oligodendrocytes to synthesize the myelin sheaths [19,20]. The cerebral cholesterol is synthesized locally through the mevalonate pathway because the blood brain barrier (BBB) prevents peripheral cholesterol from entering the central nervous system (CNS) [21]. Although cholesterol synthesis significantly decreases after completion of myelination, it continues in the adult brain at low levels, mainly in astrocytes [22], serving to modulate membrane permeability and fluidity [11] as well as the activity of neuromediator receptors and ion channels [12]. Cholesterol is particularly abundant at synapses [23], which is why even a 10% cholesterol depletion in synaptosomal membranes is able to impair the release of neurotransmitters [24]. In addition, by inactivating extracellular signal-regulated kinase (ERK) phosphorylation and downregulating the protein kinase B (PBK or Akt) pathway, cholesterol depletion could impair neuronal signaling and autophagy, as shown in cultured hippocampal neurons [25].

Nonetheless, neuronal cholesterol synthesis is crucial in the early stages of development, when glia-derived cholesterol fails to rescue embryonic neuronal death induced by the inactivation of squalene synthase [26]. In the mature brain, neurons abandon the energy-consuming process of cholesterol synthesis, relying on cholesterol delivery from astrocytes via apolipoprotein E (ApoE)-containing lipoproteins [27]. In addition, cholesterol bound to ApoE lipoproteins promotes synaptogenesis [28]. The maintenance of transcripts of the enzymes necessary for cholesterol synthesis in neurons throughout life raised interesting questions regarding their role [29]. It appears that neurons obtain intermediary molecules, such as dolichols, ubiquinones and isoprenoids, from the mevalonate pathway [30]. Isoprenoids are used for the prenylation of proteins and small GTPases, such as Ras, Rho, Rab, Sar1/Arf and Ran families, which, except for the Ran proteins and Arf proteins, which undergo myristoylation [31], need a series of post-translational modifications starting with prenylation in order to be anchored to the cytoplasmic side of membranes [30]. Especially the activity of Rho GTPases appears to be critical for axon growth and guidance, growth cone motility, dendritic arborization, as well as synapse formation [32]. Therefore, in the research setting, statin use led to a decrease in neurite outgrowth and dendritic spines density [33]. Neurons also have cholesterol 24(S)-hydroxylase encoded by the CYP46A1 gene, the knockout of which in mice has been shown to impair long-term potentiation, pointing to the essential role of cholesterol turnover for higher-order brain functions and which could be reversed by incubation of knockout mice hippocampal slides with mevalonate or geranylgeraniol [34]. Research in human subjects showed that statin treatment for 6 weeks (lovastatin, simvastatin and pravastatin at 40 mg/day) lowered both total and 24-S-hydroxycholesterol levels in the plasma of patients, not altering the ratio, but the reduction of 24-S-hydroxycholesterol surpassed 20% [35].

At the neuromuscular junction, synaptic transmission is mediated by the release of acetylcholine which acts on the plasmalemmal acetylcholine receptors (AChR). Synaptic transmission is regulated by the available number of receptors and the time spent by these receptors at the active sites of the postsynaptic membrane facing the neurotransmitter release areas [36]. The nicotinic AChR belongs to the pentameric ligand-gated ion channel receptors, a family of membrane proteins that transduce chemical signals into rapid ion fluxes at the postsynaptic membrane [37]. The lipid environment in which these proteins are embedded modulates the distribution and functional properties of the receptors [38,39]. Research has shown that cholesterol favors the organization of AChR into clusters, while cholesterol depletion induces the fragmentation of these AChR clusters and accelerates AChR endocytosis through a ligand-, dynamin-, and a clathrin-independent mechanism involving the activity of a small GTPase (Arf6) and its effectors (phospholipase D and Rac1) as opposed to inhibition of endocytosis found for most of the other membrane receptors [40,41].

## 3. 3-Hydroxy-3-Methylglutaryl-Coenzyme A (HMG-CoA) Reductases or Statins

**Figure 2:**
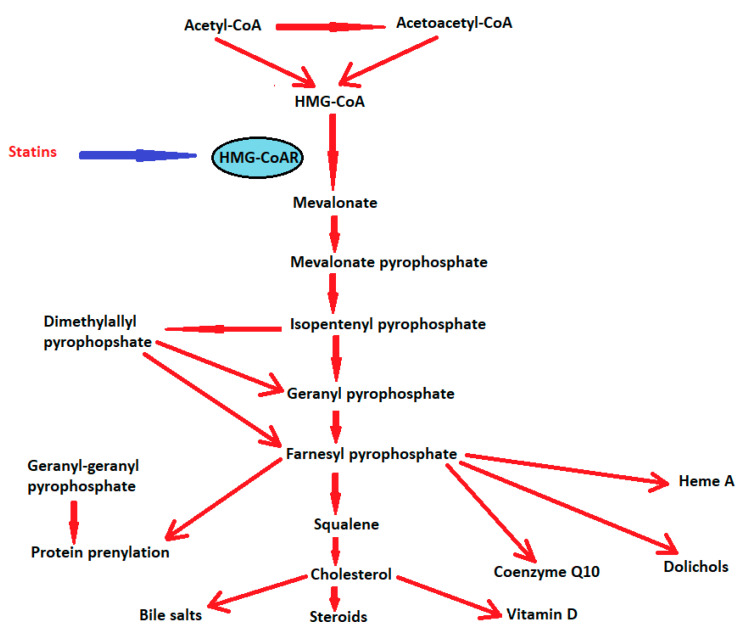
The mevalonate pathway. Statins act by inhibiting hydroxy-3-methylglutaryl-Coenzyme A reductase (HMG-CoAR), as depicted with the blue arrow.

Statins act by competitively inhibiting 3-hydroxy-3-methylglutaryl-Coenzyme A (HMG-CoA) reductase in the mevalonate pathway (Figure 2).

In response, hepatic low-density lipoprotein (LDL) receptors are upregulated [42] and bind to apolipoprotein B-rich lipoproteins facilitating their absorption by hepatocytes, thereby reducing plasmatic levels of cholesterol (mainly LDL-cholesterol) by 14–46% [43]. They also are able to modestly increase the levels of high-density lipoprotein cholesterol and lower triglycerides [44].

Aside from their cholesterol-lowering effect, statins have a series of pleiotropic effects, such as anti-inflammatory, immunomodulatory, antioxidant, atherosclerotic plaque-stabilizing, and platelet activation-inhibitory effects [45,46], through incompletely understood mechanisms.

Currently, there are 7 statins marketed: lovastatin, simvastatin, pravastatin, fluvastatin, atorvastatin, rosuvastatin, and pitavastatin [6]. While most of these molecules are lipophilic and can cross the blood brain barrier, pravastatin and rosuvastatin are hydrophilic [6]. Their metabolism also varies, leading to interindividual variability in response to therapy and a 49-fold variation in plasma concentrations of the administered statin [47]. Table 1 provides an overview of the solubility, metabolization pathways and pharmacokinetics of these statins.

Although increasingly used to prevent cardiovascular events, statins can cause adverse reactions which can significantly interfere with the patient’s quality of life, cause treatment non-adherence, or even lead to death in a small subset of patients. Among these side effects are statin-related myotoxicity (SRM) [48,49], new-onset diabetes mellitus [50], the elevation of liver enzymes [51], or memory disturbances and confusion [52]. The incidence of statin-induced adverse events differs widely depending on whether data are collected from randomized controlled trials or observational studies and also varies between countries. Muscular symptoms are reported in ranges between 2% and 10% of statin users and tend to occur mainly in persons treated with statins in an unblinded fashion [53], although up to 60% of patients who discontinued statin in the USAGE (Understanding Statin Use in America and Gaps in Patient Education Survey) study reported having discontinued therapy due to muscular side effects [54]. The risk of developing type 2 diabetes mellitus increased by 9–13% in patients taking statins [55,56], while the incidence of cognitive side effects is difficult to estimate because the trials which support statin use in cardiovascular risk reduction did not include cognitive dysfunction as a primary or secondary outcome measure [57] and due to the intricate link between cardiovascular disease and dementia [58]. A recent observational study reported that overall, 22% of patients treated with statins in the prior 2 years experienced statin-associated symptoms leading to discontinuation of therapy [59].

### 3.1. Myotoxic Effects of Statins

The myotoxic effects are most common and have been classified into 7 phenotypic categories [60], as shown in Table 2.

These effects are of various severities, but rhabdomyolysis is life-threatening and led to the voluntary withdrawal from the market of cerivastatin after 52 cases of fatal cerivastatin-induced rhabdomyolysis with kidney failure were reported [61]. The mechanism is still incompletely elucidated, but several risk factors such as increased dose, increasing age, female gender [6], Asian ancestry [62], genetic factors [63], or interactions with other drugs [64] have been identified. These factors lead to increased systemic and muscle exposure to statins, intracellular entry and disruption of muscle function [6]. It is reasonable to assume that higher systemic statin levels lead to increased statin concentrations in muscle cells because statin entry in myocytes is facilitated by several sarcolemmal transporters such as organic anion transporting polypeptide 2B1 (OATP2B1), multidrug resistance-associated protein 1 (MRP1), MRP4, MRP5, or monocarboxylate transporter-4 (MCT4) [6,65]. It appears that lipophilic statins have a greater propensity to accumulate in skeletal muscle cells than hydrophilic ones [66].

Once in the skeletal muscle fibers, statin lactones inhibit mitochondrial complex III [66], increase mitochondrial oxidative stress and interfere with the expression of pro-apoptotic genes (caspases, apoptotic protease activating factor 1—APAF1), leading to apoptosis [67,68] and reduce the circulating levels of coenzyme Q10 also competing for its pharmacodynamic target, thereby further accentuating mitochondrial dysfunction [66]. By causing the dissociation of FKBP12 (FK506 binding protein 12), a member of the FK-506 binding protein family which stabilizes ryanodine receptors in skeletal muscles, statins disturb cellular calcium homeostasis and lead to calcium release sparks [69]. Mitochondria try to buffer the excess intracellular calcium at the expense of stimulating the production of reactive oxygen and nitrogen species, which further augments mitochondrial dysfunction, a mechanism also implicated in neurodegenerative diseases [70]. In addition, statins have been shown to induce rupture of the sarcolemma and the breakdown of the T-tubular system in muscle fibers [71]. This effect has not been described after treatment with more potent PCSK9 inhibitors [72], indicating it to be rather statin-related and not caused by cholesterol depletion [6].

Statins can also induce immunologically mediated myopathies, with histologically proven necrotizing features, lymphocytic infiltration and positive anti-HMG-CoA reductase antibodies [73,74], which can persist and even progress after discontinuing statins and require immunosuppressive therapy [75]. It appears that the repair of skeletal muscle fibers requires local expansion of Foxp3-expressing CD40+ regulatory T cells (Tregs) [76]. The precise mechanisms are still under investigation, but statins have been shown to interfere with the expression of antigens in thymic stromal cells and, thereby, modulate immune tolerance [77].

### 3.2. Cognitive Side-Effects of Statins

Although most studies supporting the recommendation of statin therapy in cardiovascular prevention did not report cognitive disturbances to be a major side effect of the drugs, these trials did not list cognitive measures either as primary or as secondary outcome measures. However, post-marketing reports drew attention to transient cognitive impairment and short-term memory losses caused by statin treatment, mainly by the lipophilic simvastatin and atorvastatin, which prompted the Food and Drug Administration (FDA) to issue a warning regarding the potential for reversible cognitive impairment in statin users in 2012 [57].

Several potential mechanisms have been proposed to explain these cognitive adverse events. One such mechanism is based on the relationship between cholesterol and myelin [78]. The brain has a high cholesterol content; up to 20% of the body’s cholesterol is located in the brain [79]. Since the intact BBB prevents cholesterol uptake from external lipoproteins, cholesterol is synthesized de novo mainly in astrocytes via the mevalonate pathway [80] and is a key component of myelin. As such, inhibiting HMG-CoA interferes with the formation of myelin. In fact, in vitro and animal experiments showed simvastatin to impair the remyelination process after chemical demyelination [81,82]. In addition, limiting glial cholesterol availability significantly impairs the ability of neurons to create synapses [83], impacting the development and plasticity of the neuronal circuitry [14].

Another mechanism could be a disturbance of the function of the ion channels. As shown in recent years, several proteins, including ion channels, are regulated by cholesterol [84,85]. Ultimately, neuronal excitability is modulated by these ion channels. In vitro, simvastatin has been shown to increase neuronal excitability in cultured hippocampal cells [86], as did atorvastatin in animal experiments by diminishing the activity of G protein-gated inwardly rectifying potassium (GIRK) channels [87].

Mitochondrial dysfunction and oxidative stress are also very likely to contribute to cognitive disturbances. Statins inhibit the synthesis of mevalonate, which serves to produce both cholesterol and coenzyme Q10 [88]. Coenzyme Q10 is essential for the normal functioning of mitochondria and the production of adenine triphosphate (ATP), which is why statin-induced coenzyme Q10 depletion has been thought to be involved in excessive fatigue and myopathies [89]. Further, the inhibition of complex III of the respiratory chain by statin lactones [66] leads to increased production of reactive oxygen species and decreased antioxidant capacity [90]. Statin treatment has also been associated with reduced mitochondrial biogenesis [90], impaired mitochondrial dynamics [91], reduced calcium buffering capacity [92], and reduced mitochondrial DNA content [93]. Together, all these abnormalities may lead to increased neuronal degeneration and apoptosis, as happens in neurodegenerative diseases [70] or after ischemic stroke [94].

Statins can also modulate the transcription of several brain proteins. They have been shown to bind to peroxisome proliferator-activated receptor α (PPARα) and stimulate the expression of neurotrophins as well as enhance the expression of brain-derived neurotrophic factor (BDNF) and CREB (cAMP response element-binding protein) in the hippocampus of mice [95]. However, other researchers reported worsening of cognitive functions in animals by statins through decreasing BDNF, nerve growth factor (NGF), and serotonin levels in the brain [96,97,98]

## 4. Statins in Clinical Practice in Specific Subgroups of Patients

### 4.1. Statins in Myasthenia Gravis

Myasthenia gravis is an autoimmune disease in which autoantibodies block or destroy the nicotinic acetylcholine receptors on the sarcolemma, thereby leading to weakness of voluntary muscles of the eye, face, throat, and limbs [99]. The incidence is estimated to vary between 0.3 and 2.8 cases/per 100,000 persons [100]. The adult-onset form occurs after age 50 in men and before 40 years of age in women. Although myasthenia gravis (MG) continues to be a life-threatening condition [101], modern therapeutic approaches have led to increased survival rates, around 34 years [102], which means that MG patients have a good chance to reach the ages at which dyslipidemia becomes a prominent cardiovascular risk factor and should be addressed.

MG can also be induced by certain drugs such as D-penicillamine, immune checkpoint inhibitors, and possibly tyrosine kinase inhibitors, or aggravated by antibiotics (macrolides, fluoroquinolones, aminoglycosides, penicillins), beta-adrenergic blockers, class I antiarrhythmics, L-type calcium channel blockers, excess magnesium, and, obviously, neuromuscular blockers and inhalation anesthetics [103].

Starting in 2000, a series of case reports drew attention to the possibility of statin-induced myasthenic syndrome [99,104] or worsening of preexisting MG after statin exposure [105]. Patients with statin-induced MG were either sero-positive or-negative for acetylcholine receptor antibodies [106], but symptoms improved after statin cessation and/or immunosuppressive therapy [107] and, in some cases, reemerged after statin rechallenge [105]. In fact, by accessing the World Health Organization’s international database of suspected adverse drug reactions (VigiBase ^®^), Gras-Champel et al. identified a 2.66-fold greater odds ratio for statins in relation to the reporting of MG [103,108].

Several mechanisms have been proposed to explain the pathophysiology of these findings [108]:

- The statin’s myotoxic effects may exacerbate muscle weakness in patients with prior MG
- via depletion of coenzyme Q10, statins could induce mitochondrial dysfunction with impairment of energy production at the presynaptic membrane [109]
- statins may induce the formation of autoantibodies against acetylcholine receptors by inducing the production of cytokines [110,111]

Although an exacerbation of MG has been reported in sero-negative MG patients after ezetimibe [112], no other reports of MG worsening following PCSK9 inhibitors (alirocumab and evolocumab) or other lipid-lowering drugs such as niacin or bile acid sequestrants have been published to date [103].

### 4.2. Statins in Muscular Dystrophies

Muscular dystrophies (MD) are a group of inherited diseases in which the skeletal muscle fibers (sometimes smooth muscles and myocardium as well) progressively weaken and degenerate. The overall incidence, as well as the age of onset, varies among the different forms [113]. Muscular dystrophies can be classified according to various criteria. Broadly, we speak of 9 types of muscular dystrophies, as follows:

- Duchenne muscular dystrophy
- Becker MD
- congenital MD
- distal MD
- facioscapulohumeral MD
- Emery-Dreifuss MD
- limb-girdle MD
- oculopharyngeal MD
- myotonic MD

If Duchenne MD has a reduced lifespan, with death occurring in the patient’s fourth decade [114], patients with other forms of MD can live significantly longer, reaching ages at which cardiovascular diseases may become a significant health threat.

Myotonic MD is the most common MD among adults of European ancestry, with an incidence of about 10 cases/100,000 persons [115,116]. In addition to muscle weakness and wasting, it associates with insulin resistance [117] as well as heart conduction blocks and arrhythmias in the clinical picture [118].

Due to the myotoxic effects of statins described above, HMG-CoA reductase inhibitors can exacerbate muscle weakness and lead to increases in muscle enzyme levels if used in patients with myotonic MD to treat associated hypercholesterolemia [119]. In addition, statin-induced myotonia has also been described in animal experiments [120] as well as in human patients [121,122]. The proposed pathophysiology relies on a decrease in the chloride channel conductance, which is regulated by a calcium-phospholipid-dependent protein kinase [123]. This protein kinase could be activated by a dysfunction of the sarcoplasmic reticulum and thus lead to a decrease in chloride conductance. However, given the rarity of myotonic discharges in human patients compared to animal experiments, this mechanism has been questioned [122]. An alternative proposed mechanism involves a dysregulation in the ubiquitination pathway [124] through the upregulation of genes involved in the ubiquitin-proteasome pathway in response to myofibrillar damage [125]. Statin treatment represses the transcription of neural precursor cell expressed, developmentally downregulated-4 (NEDD4) [124], which is a negative regulator of phosphatase and tensin homologue (PTEN) [126], resulting in increased levels of PTEN and reduced PTEN ubiquitination [124]. Moreover, abnormally spliced NEDD4 isoforms identified in patients with myotonic MD [124] could further increase the susceptibility to statin-induced myotoxic effects.

Given the low prevalence of muscular dystrophies in the general population, large meta-analyses on the effect of statins in this subgroup of patients have not been published. However, case reports drew attention to statins unmasking subjacent muscular diseases [127,128,129], while in 6 out of 11 patients with a clinical picture resembling limb-girdle muscular dystrophy with creatin kinase levels exceeding 1000 IU/L and dystrophic changes on muscle biopsy but with no family history of the disease, anti-HMG-CoA reductase autoantibodies were present and could explain the clinical symptoms [130]. Moreover, by genotyping 713 patients on statin therapy, the presence of a polymorphism of the *DMPK* (rs672348) gene, which encodes a protein kinase implicated in myotonic dystrophy, as well as *COQ2* (rs4693570) encoding para-hydroxybenzoate-polyprenyltransferase, which participates in the biosynthesis of coenzyme Q10 (*p* < 0.000041) and *ATP2B1* (rs17381194) which encodes calcium transporting ATPase were all significantly linked to the development of muscular side effects [131].

### 4.3. Statins and Cognitive Impairment

Although the clinical trials leading to the approval of HMG-CoA reductase inhibitors for cardiovascular prevention described only mild confusion or euphoria as cognitive side effects of statins [132], post-marketing case reports consisted of transient and reversible short-term memory losses [57]. Due to the fact that only about 10% of drug-related adverse events are reported, it can be assumed that between 3000 and 30,000 patients have cognitive impairments every year only in the USA [133]. Subsequent studies and even meta-analyses have shown impairments in attention, working memory and the ability to learn from prior experiences after the use of statins, especially simvastatin and atorvastatin [134].

In 2013, the American College of Cardiology/American Heart Association reviewed the safety issues raised by statin therapy and concluded that the evidence on statin-induced cognitive impairment is inconsistent [57]. However, their conclusions were based mainly on the Justification for the Use of Statins in Primary Prevention (JUPITER) trial, Pravastatin in Elderly Individuals at Risk of Vascular Disease (PROSPER) trial, and Heart Protection Study (HPS) trial, which did not perform detailed neuropsychological assessments because cognitive dysfunction was neither primary nor secondary outcome measure in these studies. Moreover, PROSPER used rather low doses of pravastatin, which is a hydrophilic statin unable to cross the BBB [57].

The presumed mechanisms through which statins can lead to the cognitive impairment have been discussed above. In addition, since the cognitive disturbances appeared mainly in the elderly, increased exposure to statins due to poor metabolism can also be considered [57]. Single nucleotide polymorphisms of several genes, such as the cytochrome P450 gene family, can alter the hepatic metabolism of statins [135] and result in increased body exposure, especially since the same CYP enzymes metabolize certain antihypertensive drugs often used in elderly patients [136]. In addition, HMG-CoA reductase gene polymorphisms can influence both cholesterol-lowering effects and pleiotropic effects of statins [137], while genetic variants of the CETP (cholesteryl ester transfer protein) such as rs5882-AA or the genetic variant of NR1H2 (nuclear receptor subfamily 1 group H member 2) rs2695121-CC were associated with cognitive dysfunction, especially in patients treated with lipophilic statins [138]. As Rojas-Fernandez et al. showed, switching from a lipophilic to a hydrophilic statin can reverse acute cognitive dysfunctions [139]. Ethnic background and gender may also contribute to the different responses. The use of angiotensin receptor blockers (ARBs) and any type of statin were associated with a lower risk of AD among White patients, while no such effect was found among Black patients using rosuvastatin or Hispanic patients [136]. According to the study by Kim and colleagues [140], lovastatin decreased the risk of dementia in women, while atorvastatin reduced the risk in men. Women tend to metabolize substrates of CYP3A4 faster than men [141], and statins have been shown to inhibit estrogen receptors [142] and compete with estrogen for binding sites [141].

As for the long-term use of statins and cognition, several studies addressed this issue. In an observational study with long-term follow-up of patients who were treated with statins for a mean of 3.8 years, statin users performed worse than non-statin users. It is true that users were also older, and multiple regression analyses did not show statin use to significantly affect cognitive performance [143]. In contrast, a meta-analysis showed a beneficial role of long-term statin use in the prevention of dementia [144]. However, the link between dementia and vascular disease is complex since strategically placed strokes can significantly worsen the cognitive performances of patients, as happens with strokes damaging the angular gyrus, inferomesial temporal, mesial frontal, anterior and dorsomedial thalamus, left capsular genu or caudate nuclei [145]. As such, preventing these strokes with statin therapy could indeed prevent cognitive decline. To date, no consensus as to the usefulness of statins in the prevention of dementia or Alzheimer’s disease has been reached [57]. Moreover, research has raised the possibility of an age-dependent effect of statins on cognition, with a smaller benefit in delaying the progression of Alzheimer’s disease (AD) in people older than 65 years [146,147]. A nationwide study that divided participants into 3 age categories (65–75 years, 76–85 years, and 86 and older) and took into account also the type of statin used found that in the 65–75 age group, rosuvastatin and pravastatin reduced the risk of AD, rosuvastatin and atorvastatin decreased the same risk in the 76–85 age group, while no significant effect was found in people older than 86 years [140]. However, in a small study on ischemic stroke patients, low LDL-cholesterol levels were positively correlated with worse cognitive performances [148]. As such, future studies are needed to decipher the effects of statins in connection with aging on cognitive functions.

Comorbidities may also explain the inconsistent results of studies looking at the preventive effect of statin therapy on the risk of dementia. A renin-angiotensin system inhibitory antihypertensive drug used in combination with pravastatin or rosuvastatin was shown to reduce the risk of dementia [136], while long-term use of rosuvastatin with candesartan and hydrochlorothiazide had no effect on cognitive function [149]. The relationship between diabetes mellitus (DM) type 2 and AD is even more complicated with regard to statin use. While DM is a risk factor for AD and has been shown to accelerate cognitive decline [150] through exacerbation of oxidative stress, inflammation and atherosclerosis [151], statin therapy itself can precipitate DM, especially if used in high doses [152].

The choice of statins also influences the risk of dementia. While hydrophilic statins were associated with a lower risk of all-cause dementia, lipophilic ones lowered the risk of AD but not of vascular dementia [153].

Table 3 presents an overview of the conflicting results reported by large cohort studies and meta-analyses regarding the effect of statin treatment on the risk of developing dementia.

It is difficult to compare the various studies. Barthold et al. [136] randomly selected 20% of Medicare beneficiaries aged 67 and higher enrolled between 2007–2014 and observed for a minimum of 3 years, with statins used for at least 2 years. Of the identified 561,962 statin users, 153,120 were on atorvastatin, 77,795 on pravastatin, 62,387 were on rosuvastatin and 268,660 on simvastatin. The exact doses are not mentioned in the study. Kim et al. [140] published a retrospective cohort study on beneficiaries of the Korean Health Insurance Review and Assessment Service between 2007–2015 and included 71,587 patients who used statins for at least 6 months. Stratification was into 3 age groups, and follow-up was extended for a median of 5 years. The exact numbers of users of different statins are not given, nor the dose. Poly et al. [153] included 30 heterogeneous studies (23 cohort studies and 7 case-control studies) in a meta-analysis in which they analyzed data from a total of 9,162,509 patients, but the risk of AD was evaluated according to statin potency (atorvastatin, simvastatin and rosuvastatin being considered potent statins) and their lipo- versus hydrophilicity. Pan et al. [154] focused on patients hospitalized with stroke in Taiwan between 1997 and 2005 in their cohort study. Of the 14,807 patients identified, they randomly selected 4724 patients who were prescribed statins, which they matched with 4724 non-statin users with similar demographic characteristics and classified the statins into high and low potency and lipo- or hydrophilic ones. The cohort study of Sinyavskaya et al. [155] included 465,085 patients, of which 72% (334,861) were taking simvastatin, 20% (93,017) atorvastatin, 5% (23,254) pravastatin, 2% (9302) rosuvastatin and 1% (4651) were on fluvastatin, without further stratifying patients according to dose.

No cognitive side effects were described for the newer lipid-lowering agents, such as ezetimibe [156] or the PCSK9 inhibitor evolocumab [157], which makes them useful in patients with elevated cholesterol levels in whom cognitive dysfunction is a considerable risk or is already present.

## 5. Conclusions

HMG-CoA reductase inhibitors are important tools in reducing the risks of vascular events and cardiovascular mortality. However, physicians should be aware of the side effects and carefully balance the risks versus benefits to avoid potential life-threatening events or impairment of the patient’s quality of life. Until genetic testing is used to predict the response to statins and allow for a personalized approach in each patient, we should try to avoid trading the brain for the heart. This task is particularly difficult for neurologists who treat patients with muscular dystrophies, myasthenia gravis, or elderly patients with cognitive impairment. In order to avoid these side effects, we would suggest the following:

- using non-statin lipid-lowering therapies in patients with muscular diseases, such as ezetimibe or PCSK9 inhibitors
- extensive evaluation of patients with vascular events (ischemic strokes), with measurement of the LDL-cholesterol levels, neuropsychological evaluation, identification of the stroke subtype according to the TOAST criteria [158], and thorough risk stratification using, whenever possible, the coronary artery calcium (CAC) score [159]
- when statins are necessary, the preference for a hydrophilic statin over a lipophilic one could avoid statin-induced cognitive impairment
- the LDL-cholesterol levels should be regularly checked and the doses adjusted accordingly [148]
- However, further clinical trials may help to develop efficient therapeutic strategies and set guidelines for particular subsets of patients.

## Figures and Tables

**Table 1:** Main characteristics of marketed statins.

Statin	Dose	Solubility	Liver Metabolization	Half-Life (Hours)
Lovastatin	10–80 mg	lipophilic	CYP450 3A4	2
Fluvastatin	20–80 mg	lipophilic	CYP450 2C9	4.7
Simvastatin	5–40 mg	lipophilic	CYP4503A4	1–2
Atorvastatin	10–80 mg	lipophilic	CYP450 3A4	14
Pitavastatin	1–4 mg	lipophilic	CYP450 2C9	96
Rosuvastatin	5–40 mg	hydrophilic	CYP450 2C9 and 2C19	19
Pravastatin	20–80 mg	hydrophilic	sulphation	1–2

**Table 2:** Classification of statin-related myotoxic phenotypes (modified from Alfirevic et al., https://doi.org/10.1038/clpt.2014.121, [60]).

SRM Classification	Phenotype	Definition
SRM 0	Asymptomatic	Elevations of <4× upper normal limit in serum creatine kinase (CK)
SRM 1	Myalgia, tolerable	Muscle aches, cramps and/or weakness with no elevation of CK
SRM 2	Myalgia, intolerable	Muscle aches, cramps and/or weakness with < 4× upper normal limit of CK
SRM 3	Myopathy	CK levels > 4× but < 10× upper normal limit of CK
SRM 4	Severe myopathy	CK levels >10× but < 50× upper normal limit
SRM 5	Rhabdomyolysis	Either CK > 10× upper normal limit, muscle symptoms and renal impairment, or CK > 50× upper normal limit
SRM 6	Autoimmune-mediated, necrotizing myositis	HMGCR antibodies, HMGCR expression in muscle biopsy, incomplete resolution after statin discontinuation

CK, creatine kinase; HMGCR, 3-hydroxy-3-methylglutaryl-coenzyme A reductase; SRM, statin-related myotoxicity.

**Table 3:** Effect of statin use on the risk of developing dementia.

Solubility	Statin	Effects of Statin Use on Cognitive Function	Number of Patients	Reference
Lipophilic	Simvastatin	- significant reduction of the risk of dementia in patients with stroke - large decrease in the risk of dementia - no significant effect on the risk of dementia in patients with ischemic heart disease - associated with increased risk for Alzheimer’s disease	4724 pairs of patients 9,162,509 total number 10,888 334,861	[154] [153] [140] [155]
	Atorvastatin	- significant decrease of the risk of developing dementia in patients with stroke - reduction in the risk of dementia - decrease in the risk of dementia only in men	4724 pairs of patients 9,162,509 total number 45,753	[154] [153] [140]
	Lovastatin	- no effect on the risk for dementia - significant reduction of the risk for dementia in women with ischemic heart disease	Total of 9,162,509 567	[153] [140]
	Fluvastatin	- no effect on the risk for dementia - significant decrease in the risk of dementia in patients with stroke	Total of 9,162,509 patients 4724 pairs of patients	[153] [154]
Hydrophilic	Rosuvastatin	- significant decrease in the risk of dementia in patients with stroke - reduction of the risk for Alzheimer’s disease and related dementia if used in combination with inhibitors of the renin-angiotensin system - reduction in the dementia risk - significant reduction of the risk for dementia in patients with ischemic heart disease	4724 pairs of patients 62,387 9,162,509 8251	[154] [136] [153] [140]
	Pravastatin	- no effect on the risk for dementia - reduction of the risk for dementia in patients with ischemic heart disease - reduction of the risk for dementia if used in combination with antihypertensive drugs	9,162,509 2524 77,795	[153] [140] [136]

## Data Availability

Not applicable.
